# DO PERSONALITY TRAITS INFLUENCE THE ASSOCIATION BETWEEN DEPRESSION AND DEMENTIA IN OLD AGE?

**DOI:** 10.1093/geroni/igac059.527

**Published:** 2022-12-20

**Authors:** Khuzeman Abbasi, Ujunwa Ebili, Henry Egbuchiem, Afolabi Orekoya, Olawale Olanisa, Abimbola Arisoyin, Adeleye Adaralegbe

**Affiliations:** Vanderbilt University Medical Center, Glen Burnie, Maryland, United States; Emel Specialists and Pediatric Center, Festac Town, Lagos, Nigeria; The Everest Foundation, Los Angeles, California, United States; Wellspring Medical Center, Riverdale, Georgia, United States; IQVIA Biotech, Morrisville, North Carolina, United States; TCA health, Lagos, Lagos, Nigeria; University of Connecticut, Storrs, Connecticut, United States

## Abstract

Depression and personality traits are independent predictors of dementia or cognitive impairment. Despite the well-established relationship between these two psychosocial factors and dementia, no research has been documented on how personality traits can influence dementia in older adults exhibiting depressive symptoms. This study explored the influence of personality traits on the association between change in depression and dementia in old age. We used a population-based longitudinal cohort study involving two waves of data collected 5 years apart, containing 2210 American older adults, from the National Social Life, Health, and Aging Project to explore if personality traits influence how change in depression predicts the development of dementia. We used multivariate logistic regression to examine the relationship between depression and dementia at T2 while adjusting for sociodemographic characteristics, comorbidity index at T1, and baseline dementia. Change in depression increased the likelihood of dementia at T2 by 4.2% (AOR = 1.04, p = 0.019) in the co-variate adjusted model. Personality traits, overall, did not influence how depression predicts the development of dementia. However, agreeableness individually nullified the effect of depression on the development of dementia, whereas extraversion was the only personality trait that significantly predicted dementia. We recommend the promotion of prosocial behaviors in old age as these appear to be protective. In addition, early life education and a strong social support can keep the depression-dementia spectrum at bay in old age.

